# Data-independent LC-MS/MS analysis of ME/CFS plasma reveals a dysregulated coagulation system, endothelial dysfunction, downregulation of complement machinery

**DOI:** 10.1186/s12933-024-02315-x

**Published:** 2024-07-16

**Authors:** Massimo Nunes, Mare Vlok, Amy Proal, Douglas B. Kell, Etheresia Pretorius

**Affiliations:** 1 Department of Physiological Sciences, Faculty of Science, Stellenbosch University, Private Bag X1 Matieland, Stellenbosch, 7602 South Africa; 2 Central Analytical Facility: Mass Spectrometry, Stellenbosch University, Tygerberg Campus, Room 6054, Clinical Building, Francie Van Zijl Drive Tygerberg, Cape Town, 7505 South Africa; 3 PolyBio Research Foundation, 7900 SE 28th ST, Suite 412, Mercer Island, DC 98040 USA; 4 Department of Biochemistry and Systems Biology, Institute of Systems, Molecular and Integrative Biology, Faculty of Health and Life Sciences, University of Liverpool, Crown St, Liverpool, L69 7ZB UK; 5 The Novo Nordisk Foundation Centre for Biosustainability, Technical University of Denmark, Building 220, Chemitorvet 200, 2800 Kongens Lyngby, Denmark

**Keywords:** Myalgic encephalomyelitis/Chronic fatigue syndrome (ME/CFS), Proteomics, Thrombotic pathology, Endothelial pathology

## Abstract

Myalgic encephalomyelitis/chronic fatigue syndrome (ME/CFS) is a debilitating chronic condition that is characterized by unresolved fatigue, post-exertion symptom exacerbation (PESE), cognitive dysfunction, orthostatic intolerance, and other symptoms. ME/CFS lacks established clinical biomarkers and requires further elucidation of disease mechanisms. A growing number of studies demonstrate signs of hematological and cardiovascular pathology in ME/CFS cohorts, including hyperactivated platelets, endothelial dysfunction, vascular dysregulation, and anomalous clotting processes. To build on these findings, and to identify potential biomarkers that can be related to pathophysiology, we measured differences in protein expression in platelet-poor plasma (PPP) samples from 15 ME/CFS study participants and 10 controls not previously infected with SARS-CoV-2, using DIA LC-MS/MS. We identified 24 proteins that are significantly increased in the ME/CFS group compared to the controls, and 21 proteins that are significantly downregulated. Proteins related to clotting processes – thrombospondin-1 (important in platelet activation), platelet factor 4, and protein S – were differentially expressed in the ME/CFS group, suggestive of a dysregulated coagulation system and abnormal endothelial function. Complement machinery was also significantly downregulated, including C9 which forms part of the membrane attack complex. Additionally, we identified a significant upregulation of lactotransferrin, protein S100-A9, and an immunoglobulin variant. The findings from this experiment further implicate the coagulation and immune system in ME/CFS, and bring to attention the pathology of or imposed on the endothelium. This study highlights potential systems and proteins that require further research with regards to their contribution to the pathogenesis of ME/CFS, symptom manifestation, and biomarker potential, and also gives insight into the hematological and cardiovascular risk for ME/CFS individuals affected by diabetes mellitus.

**Graphical abstract:**

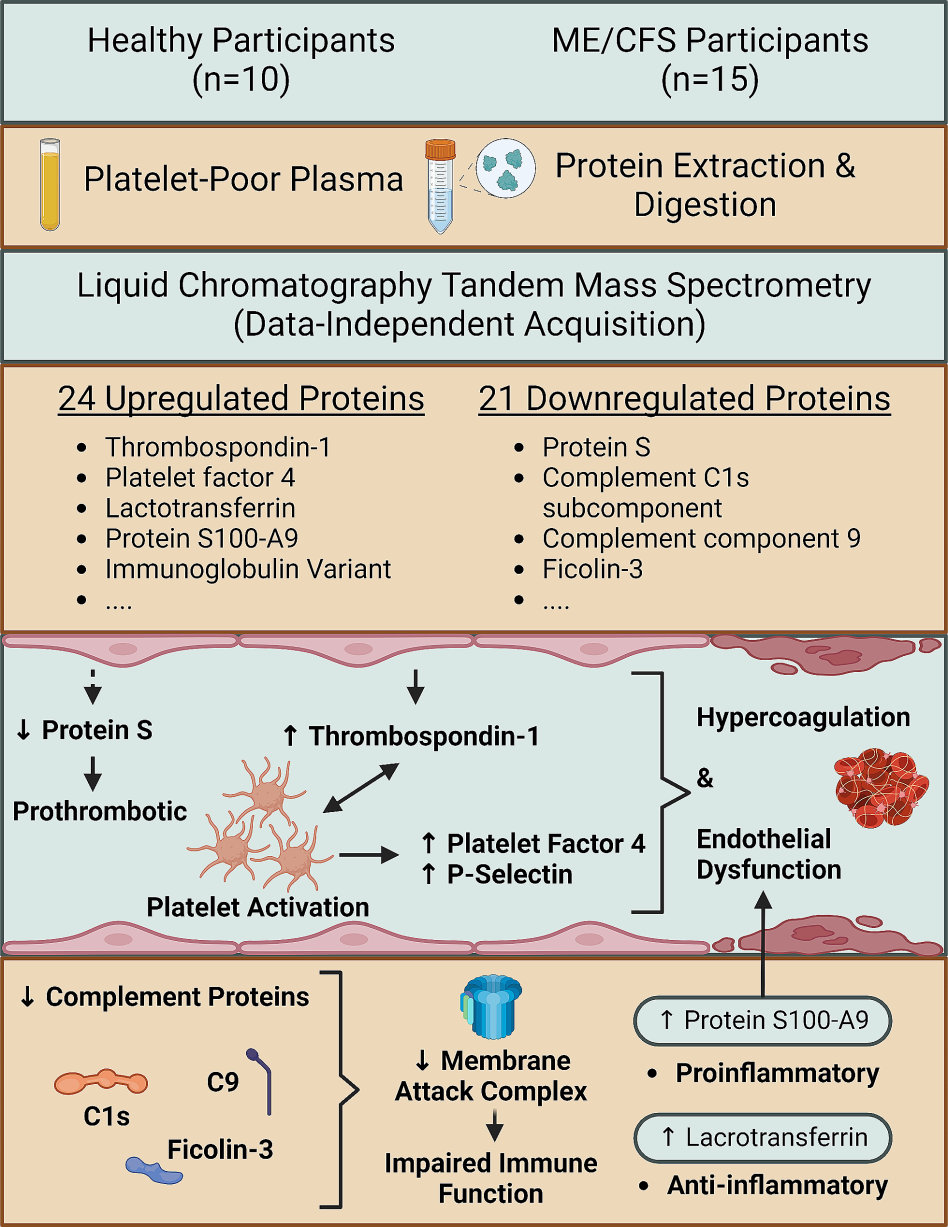

**Supplementary Information:**

The online version contains supplementary material available at 10.1186/s12933-024-02315-x.

## Introduction

Myalgic encephalomyelitis/chronic fatigue syndrome (ME/CFS) is a debilitating chronic condition that manifests in various physiological systems and is characterized by unresolved fatigue and post-exertional symptom exacerbation (PESE) [1, 2]. The onset of ME/CFS is often connected to viral/bacterial infection [3, 4], with the herpesviruses being most implicated [5–7]. However, there are no widely-established clinical biomarkers for the condition and much of the disease pathogenesis remains unknown.

We have previously shown that whole blood and platelet-poor plasma (PPP) from ME/CFS study participants showed hypercoagulability as measured via thromboelastography and platelet visualization [8]. PPP from the ME/CFS group also contained significant levels of amyloid fibrin(ogen) (some 10× in area of that of the controls), albeit less and of smaller size than observed in Long COVID PPP samples [9, 10].

Along with our study, a growing body of research suggests that cardiovascular and hematological abnormalities, such as endothelial dysfunction [11–18], abnormal blood flow and hence vascular dysregulation [19–21], and hyperactivated platelets [8, 22, 23] may contribute to the ME/CFS disease process [24]. Furthermore, it has been demonstrated that plasma from ME/CFS individuals cause dysfunction of healthy endothelial cells [12], and prompts the assessment of the blood for potential diagnostic and mechanism-related biomarkers.

To expand on these findings, we randomly selected a subset of ME/CFS and healthy control PPP samples from our previous study cohort and used data-independent acquisition (DIA) mass spectrometry (MS) (DIA LC-MS/MS) to identify differentially expressed proteins. DIA LC-MS/MS possesses the capabilities to capture nearly the entire proteome of a sample, enabling global and nominally unbiased detection and quantification of peptides [25]. Using this untargeted approach, we identified statistically significant differences in the levels of 45 proteins between ME/CFS and healthy controls. A select few came with both strong statistical significance and exhibit compelling fold changes, and, consistent with other data [8, 16, 18, 22, 23, 26], these pertain to the endothelium and coagulation system, and immune function.

## Methods

### Ethical statement

Ethical clearance was issued by the Health Research Ethics Committee (HREC) of Stellenbosch University (South Africa) (N19/03/043, project ID #9521). Strict compliance to ethical guidelines were carried out, as guided by the Declaration of Helsinki, South African Guidelines for Good Clinical Practice, and Medical Research Council Ethical Guidelines for Research.

### Blood collection and demographics

Stored PPP samples from 10 healthy individuals were used as controls for this study. The exclusion principles applied to the controls include smoking, pregnancy, contraceptives, cardiovascular disease, coagulopathy, and a previous SARS-CoV-2 infection. 15 stored ME/CFS PPP samples, which were a part of a larger sample group that was collected for a previous study [8], were included in this experiment. ME/CFS blood samples were recruited via the ME/CFS Foundation of South Africa. All ME/CFS participants in this study had not experienced a SARS-CoV-2 infection prior to the date of sample collection. Sodium citrate tubes were used for blood collection, and centrifuged at 3000×g for 15 min at room temperature. The PPP was collected, and stored at –80 °C. ME/CFS individuals were asked to complete the ICC Symptom Questionnaire [27] to further describe the types and severity of symptoms experienced by this cohort.

### Sample preparation for proteomics

10 control and 15 ME/CFS stored platelet-poor plasma (PPP) samples were used for the proteomics analysis. Protein determination was performed using a Jenway 7415 Nano Micro-Volume Spectrophotometer. Samples were diluted 20× with ammonium bicarbonate. 50 µg of protein was obtained from each sample and the volume was readjusted to 50 µL with Tris-Buffer (0.1 M; 1% DS). Disulphide bridges were reduced using 5 mM TCEP (tris(2-carboxyethyl)phosphine) at room temperature for 1 h. Cysteine residues were then blocked with 10mM MMTS (Methyl methanethiosulfonate) for 30 min at room temperature. Interfering substances were removed prior to digestion by washing on bead using MagResyn HILIC (https://resynbio.com/wp-content/uploads/2019/12/IFU_HILIC.pdf). Samples were digested on MagResyn HILIC particles with 1 µg trypsin (Pierce) at an enzyme: substrate of 1:50. Samples were left to incubate for 18 h at 37 °C. The supernatant was then collected, and the MagResyn HILIC particles were washed with 50 µL 1% TFA (trifluoroacetic acid) to remove any peptides bound to the particles. This supernatant was then also collected. Supernatants were combined, dried, and the peptides were resuspended in 50µL of 50% acetonitrile and then centrifuged at 10,000g for 5 min to remove any particulate. 20 µL of supernatant was then removed and dried. The samples were resuspended in loading solvent (See Liquid Chromatography section below) containing Biognosys 11 iRTs (indexed retention time standards) at 0.05/µL in preparation for mass spectrometry.

### Liquid chromatography (Dionex nano-RSLC)

Liquid chromatography experiments were conducted using the Thermo Scientific Ultimate 3000 RSLC equipped with a 20 mm × 100 μm C18 trap column (Thermo Scientific) and a CSH 25 cm × 75 μm, 1.7 μm particle size C18 column (Waters) analytical column. The loading solvent was constituted by 2% acetonitrile: water and 0.1% formic acid; Solvent A: water and 0.1% formic acid; and Solvent B: 100% acetonitrile containing 0.1% formic acid. Samples were transferred onto the trap column from an autosampler (set to 7 °C) at a flow rate of 2µL/min, for 5 min prior to the samples being eluted onto the analytical column. 300nL/min defined the flow rate, and the gradient occurred as follows: 5–30% B over 60 min and 30–50% B from 60 to 80 min. The experiment was performed at 45 °C.

### Library building: data-dependent acquisition (DDA)

Samples were pooled for the construction of the library, to obtain a complete (as possible) protein database from our samples. Samples were reduced and cysteine residues blocked as described above, and the MagResyn HILIC protocol (https://resynbio.com/wp-content/uploads/2019/12/IFU_HILIC.pdf) was again used for protein clean-up. Protein digest was performed on MagResyn HILIC particles with 1 µg trypsin as previously described. A peptide clean-up was performed using HILIC Clean-Up of Peptides Post Protein Digestion (https://resynbio.com/wp-content/uploads/2021/12/HILIC_PEPCLU.pdf). Samples were then cleaned and further fractionated using Pierce^™^ C18 Spin Tips & Columns (catalogue number: 89,870) with acetonitrile (50%, 20%, 17.5%, 15%, 12.5%, 10%, 7.5%, and 5%). LC experiments were carried out as described previously. The mass spectrometry analysis was performed using a Thermo Scientifc Fusion mass spectrometer equipped with a Nanospray Flex ionization source. Positive mode was chosen with spray voltage set to 2 kV and the ion transfer capillary set to 290 °C. Internal calibration of spectra was conducted using polysiloxane ions at m/z = 445.12003. MS1 scans were performed using the Orbitrap detector set at 60,000 resolution over the scan range 375–1500 with AGC target at 4E5, and maximum injection time of 50 ms. Data was acquired in profile mode. MS2 acquisitions were carried out using monoisotopic precursor selection for ion with charges + 2 to +7 with error tolerance set to ± 10 ppm. Precursor ions were excluded from fragmentation once for a period of 60 s. Precursor ions were selected for fragmentation in HCD mode using the quadrupole mass analyser with HCD energy set to 30%. Fragment ions were detected in the Orbitrap mass analyzer set to 30,000 resolution. The AGC target was set to 5E4 and the maximum injection time to 60 ms. The data was acquired in centroid mode.

### Mass spectrometry: data independent acquisition (DIA)/SWATH

A Thermo Scientifc Fusion mass spectrometer equipped with a Nanospray Flex ionization source was used in this study, as previously mentioned. Samples entered via a stainless-steel nano-bore emitter. Positive mode was used for data collection with the spray voltage set to 2 kV and ion transfer capillary set at 290 °C. Alignment of chromatograms were done with the aid of the iRTs kit (Biognosys). MS1 scans were performed using the Orbitrap detector set at 60,000 resolution over a m/z range of 375–1500. The automatic gain control (AGC) target was set to standard and maximum injection time at 100 ms. Data were acquired in profile mode. Precursor ions were selected for fragmentation in higher-energy C-trap dissociation (HCD) mode using the quadrupole mass analyzer with HCD energy set to 30%. Precursor ions were scanned in three windows, 355–555, 555–755, and 755–955 m/z (which were saved as three separate raw files), with an isolation window of 10 m/z and an overlap of 1 m/z. Ions were detected in the Orbitrap mass analyzer set to 30,000 resolution. The AGC target was set to custom and the maximum injection time mode set to custom. The data were acquired in centroid mode.

### Data analysis

The raw files generated by the mass spectrometer were imported into Skyline (version 22.2.0.312) using the DIA wizard. Precursor and ion charges were set to 2, 3 4 and 1, 2, 3, respectively, and shuffle sequence chosen as the decoy generation method. Semi-tryptic cleavage with 1 missed cleavage was allowed for. Precursor mass tolerance was set to 10 ppm and fragment mass tolerance set to 0.02 Da. Deamidation (NQ), oxidation (M), and methylthio (C) were allowed as dynamic modifications. Equalize medians was chosen as the normalization method with a 95% confidence interval. mProphet, which automatically adapts the error model for each data set and assigns a confidence measure to each peak group for quality control, was included in the analysis to score peptide identifications using its linear model [28]. A Q value of 0.05 was chosen. A database was constructed from UniProt using the keywords ‘plasma’, ‘immune system’, and ‘herpesviruses’. We also ran the analysis against the spectral library created from patient samples.

Quality control plots for all proteins (to assess system performance) were obtained using MSstatsShiny, whereby the Skyline output and annotation files, after rearranging the layout, were used as MSstatsShiny input files. Proteins with only 1 feature were not removed from the analysis, and a Q value of 0.05 was selected. For normalization, equalize medians were chosen. Missing values were censored and model-based imputation utilized. Runs with over 50% missing values were also removed.

## Results

**Fig. 1:**
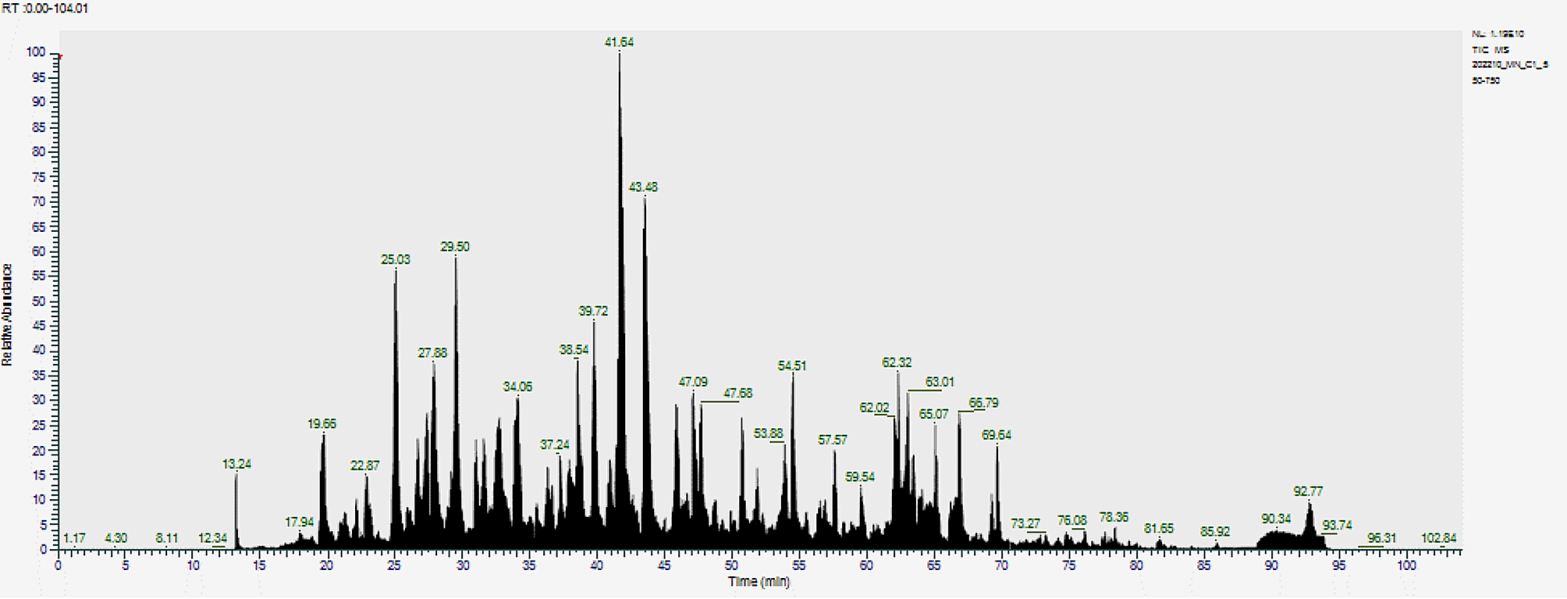
Total Ion Chromatogram (TIC)

**Fig. 2:**
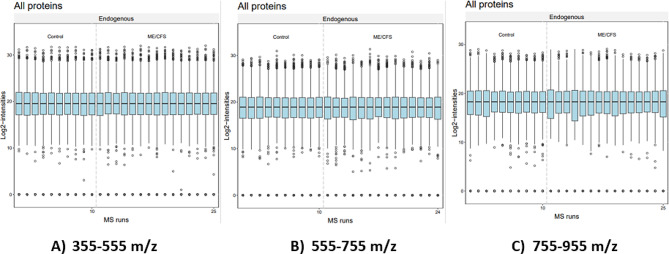
Quality control plots of protein levels of all samples across the three m/z ranges. Data were processed on MSstatsShiny. Normalization was performed using equalize medians

The demographics of the participant groups are contained within Table 1, along with the symptom severity score averages [27] of the ME/CFS population. A portion of the ME/CFS cohort presents with symptoms and comorbidities that are intimately associated with ME/CFS, specifically gastrointestinal issues (6/15 participants) [29], orthostatic symptoms (5/15 participants) [30–32], and fibromyalgia (3/15 participants) [33]. Proteomics data from the analysis of control (*n* = 10) and ME/CFS (*n* = 15) PPP using Skyline is represented in Table 2 and the total ion chromatogram (TIC) is depicted in Fig. 1. Figure 2 is a representation of the quality plots for all proteins/peptides across the three m/z ranges, which was obtained from MSstatsShiny. Our experiment indicates that 24 proteins are significantly increased in the ME/CFS group compared to the controls, and that 21 proteins are significantly downregulated. However, only a select few hold strong statistical significance. These will be presented and discussed, while the other identified protein data can be perused in Supplementary Material 1. In some cases, only a limited number of peptides from a protein are significant, which may reflect regulated peptides or be related to the number of ions entering the mass spectrometer at a given time point. The transitions for proteins detected by a single peptide are contained within Supplementary Material 2.

**Table 1:** Demographics of the ME/CFS cohort and symptom score averages for the ICC questionnaire

Age	
Age of control population (n=10; 7 females)	59.3 ± 7.5
Age of ME/CFS population (n=15; 11 females)	48.9 ± 14.9
P Value (parametric)	0.054
Comorbidities of ME/CFS Population (%)	
Gastrointestinal Symptoms	40
POTS	33
Gingivitis/Periodontitis	20
Hypercholesterolemia	20
Fibromyalgia	20
Psoriasis	13
Rheumatoid Arthritis	13
Hypertension	13
Mast Cell Activation Syndrome	7
Rosacea	7
ICC Questionnaire Results of ME/CFS Population (mean SD)	
Post-Exertional Neuroimmune exhaustion	7.7 ± 1.9
Neurological Impairments	6.5 ± 2.8
Immuno, Gastrointestinal, and Genitourinary impairments	5.9 ± 2.8
Energy Production/Transportation Impairments	6.5 ± 2.8

The ICC questionnaire and the comorbidities are both self-reported by the patients

Statistical significance was determined at *p* < 0.05

Data are represented as mean ± SD

**Table 2:** Selected significant protein and peptide data from the Skyline analysis (all data can be found in Supplementary Material 1)

Protein name	Peptide sequence	Fold Change (ME/CFS raised)	*p* value	UniProt accession number	No. of peptides	m/z range
Thrombospondin-1	GPDPSSPAFR	3.55	0.00009	P07996	2	355–555 m/z
	TIVTTLQDSIR	3.48	0.0002	P07996	2	555–755 m/z
	IEDANLIPPVPDDKFQDLVDAVR	3.75	0.00009	P07996	3	755–955 m/z
Platelet factor 4	HITSLEVIK	3.11	0.00009	P02776	1	355–555 m/z
Vitamin K-dependent protein S	QSTNAYPDLR	0.48	0.0006	P07225	2	555–755 m/z
Complement C1s subcomponent	MLTPEHVFIHPGWK	0.7	0.0069	P09871	7	555–755 m/z
	MLTPEHVFIHPGWK	0.53	0.0013	P09871	5	755–955 m/z
Complement component C9	ISEGLPALEFPNEK	0.17	0.0001	P02748	6	755–955 m/z
Ficolin-3	YGIDWASGR	0.65	0.0006	O75636	5	355–555 m/z
	QDGSVDFFR	0.45	0.0086	O75636	5	355–555 m/z
	LLGEVDHYQLALGK	0.49	0.0348	O75636	5	355–555 m/z
	LLGEVDHYQLALGK	0.53	0.0084	O75636	1	755–955 m/z
Lactotransferrin	LRPVAAEVYGTER	7.05	0.00009	P02788	5	355–555 m/z
	FQLFGSPSGQK	8.38	0.00009	P02788	3	555–755 m/z
	IDSGLYLGSGYFTAIQNLR	4.52	0.0118	P02788	3	555–755 m/z
Protein S100-A9	NIETIINTFHQYSVK	2.08	0.0159	P06702	1	555–755 m/z
	NIETIINTFHQYSVK	2.89	0.0046	P06702	1	755–955 m/z
Immunoglobulin heavy constant gamma 1	DTLMISR	1.51	0.0094	P01857	6	355–555 m/z
	GFYPSDIAVEWESNGQPENNYK	5.64	0.0031	P01857	20	755–955 m/z

Fold change is expressed with reference to the ME/CFS group

Significance was determined at *p* < 0.05

## Discussion

In the present study, we employed DIA LC-MS/MS to search for any significant differences in protein levels between control and ME/CFS blood (PPP) samples. Albeit a small sample size, we offer insight into the differential protein levels between ME/CFS and control cohorts, and provide direction for future studies with larger cohorts. We identified 45 proteins whose differences in expression level are statistically significant (*p* < 0.05), but only a select few – the ones with strong statistical significance and a notable fold change – are discussed here.

### Proteins related to the coagulation system

We identified three significant proteins related to the coagulation system that deserves discussion: thrombospondin-1; platelet factor 4; and vitamin K-dependent protein S. When interpreting these findings, there are a number of recently published articles implicating platelets and other components of and processes related to the coagulation system in ME/CFS cohorts [8, 22–24, 34, 35], as well as endothelial dysfunction [11–14, 16–18, 36]. The links between platelets, coagulation, and endothelial cells, and dysfunction thereof are discussed elsewhere [37–39]. Further work is now required to elucidate the impact of clotting and endothelial dysfunction on ME/CFS pathology and symptom presentation, and determine if any treatment can result from these findings.

Thrombospondin-1 (TSP-1) is a glycoprotein, part of a family of 5 thrombospondins [40], that is involved in platelet activation [41, 42], clot formation [43–45], haemostasis [46], vascular control [47, 48], inflammation [49, 50], and tissue repair [51]. TSP-1 is found within the extracellular matrix [52], α-granules of platelets [53], endothelial cells and macrophages [51, 54], and plasma [55]. Elevations in TSP-1 induce endothelial dysfunction and interfere with vascular control via a number of mechanisms, including modulation of nitric oxide [47, 48, 56–58]. The potential of TSP-1 to influence vascular control might have relevance to impaired blood flow observed in ME/CFS [19, 59]. In the context of platelets, TSP-1 leads to platelet activation via signalling of inhibitory cyclic adenosine monophosphate (cAMP) [46]. Furthermore, TSP-1-deficient mice exhibit signs of excessive bleeding and impaired coagulation [46]. The researchers also discovered that transfusion of wild-type platelets into TSP-1^−/−^ mice improved clot formation and stability. Hence, TSP-1 plays an important role in platelet function and clot formation.

The exact cellular source of TSP-1 in our ME/CFS population is uncertain, as immune cells [60] and endothelial cells [54] are capable of increasing plasma levels of this protein. The increase in TSP-1 in our ME/CFS cohort might be related to the active states of platelets observed in a subset of these individuals [8, 22, 23], and might help explain endothelial dysregulation and impaired vascular control in this disease population. Interestingly, TSP-1 levels increase and originate from activated platelets during SARS-CoV-2 infection [61].

Platelet factor 4 (PF4) is a CXC chemokine that, much like TSP-1 (and P-selectin), is released from α-granules of activated platelets [62, 63]. Although we only detected a single peptide for PF4 (transition depicted in Supplementary Material 2), its presence in excess is expected when one reviews the evidence of platelet hyperactivity in ME/CFS [8, 22, 23, 35] and related chronic, inflammatory diseases [64]. Its primary function is to facilitate coagulation by neutralizing glycosaminoglycans on endothelial and platelet membranes, prompting platelet aggregation and monocyte recruitment [65–67]. In addition to clotting-specific functions, PF4 also seems to be involved in immune functioning, with its secretion and serum levels increasing during infection – which is expected as platelet activation increases in response to infection [68–70]. PF4 is protective against numerous microorganisms and exerts notable antiviral effects [70–73].

PF4 has been implicated in cardiovascular disease [74–76] and gastrointestinal conditions [77, 78], and has the potential to promote oxidative and nitrosative stress, and subsequent inflammatory sequelae [79–81], although some studies argue otherwise [82]. PF4 modulates endothelial and vascular smooth muscle cells in a proinflammatory manner [66, 83] and is likely an ongoing consequence of platelet hyperactivity in ME/CFS (along with elevations in TSP-1), potentially accounting for signs of endothelial damage observed in patients [12, 16, 18]. In Long COVID, plasma PF4 levels are significantly increased compared to controls [84].

Vitamin K-dependent protein S (PROS) is an endogenous anticoagulant which exists both free in plasma and as a complex where it is non-covalently bound to complement C4-B [85, 86], which, interestingly, is significantly downregulated in the ME/CFS group. PROS functions as a cofactor to another endogenous anticoagulant, protein C, and is required for the inactivation of activated clotting factor VIII [87]. Deficiencies of PROS leads to overzealous clot formation which confers an increased risk for venous thrombosis and, in severe cases, death [86]. Apart from its role in coagulation, PROS is also involved in bone metabolism [88, 89]. Within the hematological system, endothelial cells – a cell-type intimately involved in the regulation of hemostasis – are known to secrete PROS [90]. The downregulation of PROS in the ME/CFS group, along with increases in TSP-1 and PF4, further emphasize a procoagulant phenotype in this ME/CFS population, as well as dysregulated endothelial function.

**Fig. 3:**
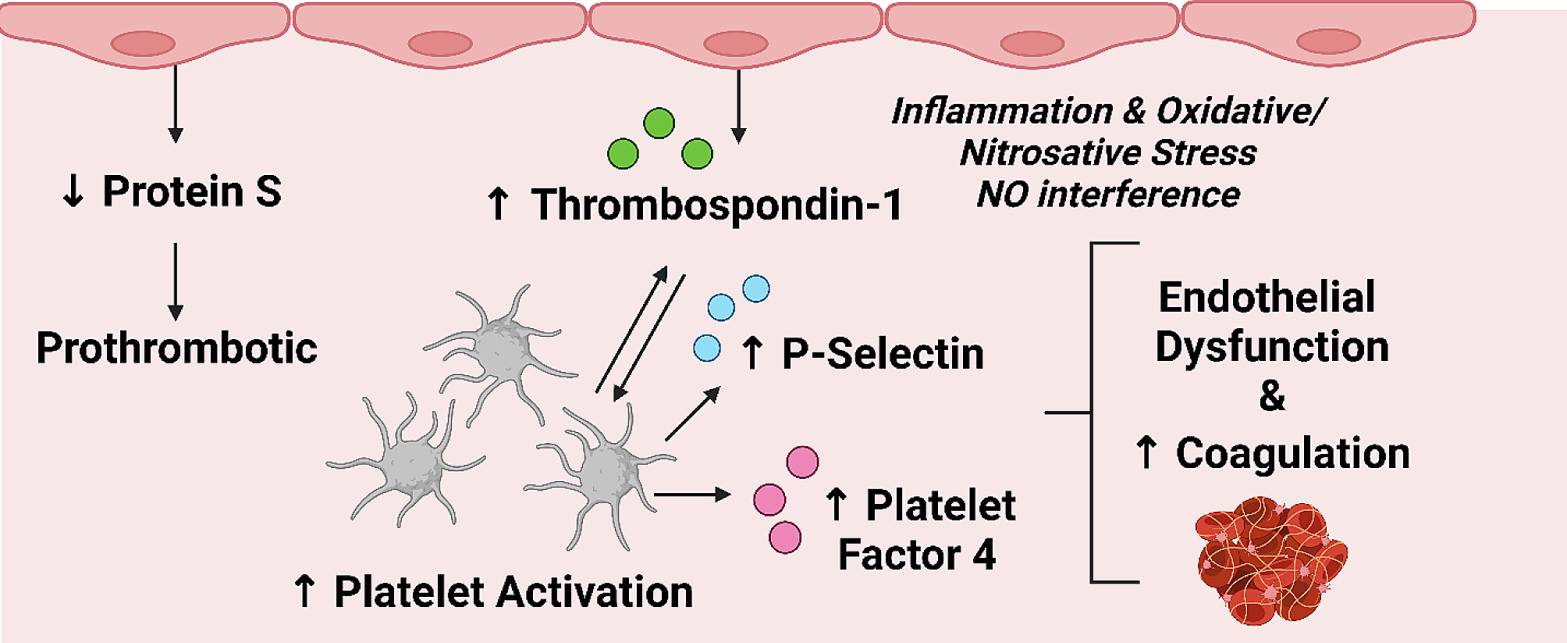
Representation of the dysregulated coagulation system in the ME/CFS group as inferred from the results of this study (Table 2). The ME/CFS group exhibits an increased propensity for clotting due to decreased levels of vitamin K-dependent protein S (an endogenous anticoagulant). Increased levels of thrombospondin-1 (originating from endothelial cells and platelets) activate platelets, as well as contribute to endothelial dysfunction via proinflammatory and oxidative/nitrosative mechanisms – as does increased levels of platelet factor 4 and P-selectin [8]. The end result is a prothrombotic state, potentially resulting from and contributing to endothelial dysfunction

Besides the decreased anticoagulant capacity conferred by decreased levels of PROS, fibrinolysis may also be hindered as PROS facilitates clot degradation via a protein C-dependent mechanism [91]. Relevant to this finding of decreased PROS in the ME/CFS group, individuals suffering from acute COVID-19 and presenting with respiratory distress exhibit decreased serum levels of PROS [92], which likely contribute to the procoagulant pathology associated with SARS-CoV-2 infection [93, 94]. Furthermore, a proteomics analysis of extracellular vesicles obtained from ME/CFS plasma samples identified high levels of SERPINA5, which is involved in hemostasis, particularly by inhibiting protein C [95]. This finding is also indicative of a prothrombotic tendency that occurs by regulating the activity of protein C. A summary of these coagulation-related findings is given within Fig. 3.

### Proteins related to the Immune System and inflammation

Leukocyte dysfunction [96–99] and dysregulated inflammatory processes [100–106] are documented characteristics of ME/CFS. While we did not identify any significant differences between the two groups with regards to notorious proinflammatory cytokines, such as TNF-α, NF-κB, and IL-1β, we did identify a dysregulation of complement factors and other inflammation-related proteins, including lactotransferrin, protein S100-A9, and an immunoglobulin variant.

The complement system is a well-established element of the innate immune system, with more recent studies revealing its participation in adaptive immunity [107]. There are three different pathways, namely the classical, lecithin, and alternative pathway, which are discussed elsewhere [108]. Dysregulation of the complement system has been documented in both COVID-19 [109] and ME/CFS [110–113].

A previous study showed that a subgroup of ME/CFS individuals (107/250) expressed significantly higher levels of complement factor C1q [110], which, in our study, is not significantly different between groups. Rather, a subunit of the C1 complex, C1s, is downregulated in the ME/CFS group. C9 forms part of the membrane attack complex (or C5b-9) that is used to lyse targeted cells [114, 115], and C9 also contributes to inflammasome activation during infection [116]. In the ME/CFS group this complement protein is significantly downregulated, exhibiting a fold change of 0.17. Impaired complement function characterized by a reduced capacity to form membrane attack complexes and aid in inflammasome activation will certainly result in shortcomings in immune defence.

Ficolin-3 is a pattern recognition receptor which functions within the lectin complement pathway and exerts antibacterial and antiviral effects [117]. Importantly, deficiency of ficolin-3 results in immunodeficiency [118] and is associated with an higher risk (8-fold) of developing a disease and autoimmunity [119]. What may be of relevance is that ficolin dysfunction or under-expression is associated with viral infection and disease [117], and hence may have a major role to play in the ME/CFS disease process [3, 5], related to herpesviruses and other microorganisms. In contrast to our finding of decreased ficolin-3 levels, a previous study found an increase in ficolin-3 expression in leukocytes from ME/CFS patients [120].

The downregulation of these complement proteins corroborates the notion of immune dysfunction in ME/CFS and may confer a susceptibility to infections, and perhaps contribute to the symptoms of malaise and fatigue [1]. Furthermore, viruses are known to have evolved strategies to bypass host defences, and the complement system is a target of such evasive processes [108]. It is speculative, but plausible to propose that these results are a consequence of such viral infection and subsequent maladaptation of the immune system. Further investigation of complement function in ME/CFS is required.

Lactotransferrin (LF) is a non-hematic iron-binding, pleiotropic glycoprotein that is found in mammalian milk, and is produced by a variety of cells, including immune cells [121, 122]. LF, apart from its ability to bind Fe^3+^ ions and prevent Fe^3+^-induced oxidative stress and inflammation [123–125], is well known for its antimicrobial, antioxidant, anti-inflammatory, prebiotic, and probiotic effects, and, hence, therapeutic potential [122, 126–129].

The physiological protection offered by LF extends into multiple organ systems, especially the immune system. It acts as a mediator of immune function, whereby, apart from enhancing certain aspects inflammation, serves to prevent an exaggerated inflammatory response and subsequent tissue damage [130–132]. It exerts chemotactic effects on leukocytes [133] and prevents the release of proinflammatory cytokines [134], likely by inhibiting TLR4 activity [135]. Due to its antimicrobial and immunomodulatory effects, LF forms part of the innate defence, and even bridges components of the innate branch to the acquired branch of the immune system [130, 133, 136].

As a biomarker, LF is useful at monitoring inflammation [137–139]. In our ME/CFS population, the increase in serum LF might be an indicator of ongoing inflammation, and even an attempt to counter proinflammatory processes associated with pathology. With speculation aside, the large fold change in this protein might be suggestive of reason for further investigation.

Protein S100-A9 – not to be confused with the endogenous anticoagulant, protein S – is a member of the S100 protein family and is known for its role in inflammation [140]. S100-A9 is predominantly expressed by immune cells, such as monocytes, macrophages, and neutrophils [141, 142], and is recognised as a damage-associated molecular pattern molecule and antimicrobial involved in the innate response [143–145]. Additionally, it acts as a chemotactic agent for phagocytes [146, 147] and is also essential for the translocation of leukocytes across the endothelium, due to its role in microtubule reorganisation [148].

S100-A9 is upregulated during infection, inflammation, and disease, and is highly expressed at sites of inflammation and injury [149–155]. Studies have shown that S100-A9 is proinflammatory, disrupts endocrine signalling, activates NF-κB, and interacts with TLR4 and the receptor for advanced glycation end products [146, 156–160]. Overexpression of S100-A9 can be more damaging than beneficial, as it can result in overzealous immune activity and subsequent inflammatory and oxidative damage, and even toxic shock [157].

S100-A9 promotes a proinflammatory and prothrombotic phenotype in endothelial cells, impairs cell-adhesion processes of the endothelium (thereby increasing vascular permeability, which coincides with the function of leukocyte recruitment and migration), and upregulates endothelial TSP-1 expression [143, 161] – a phenomenon which perhaps underlies the increase in TSP-1 exhibited by the ME/CFS group in this study. The potential of S100-A9 to cause endothelial dysfunction and damage, as well as its overexpression during inflammatory states and its proinflammatory nature, may be of relevance to ME/CFS, a disease which is characterized by endothelial dysfunction [11, 12, 14, 16, 17, 162] and (dysregulated) inflammation [26, 101, 104, 106]. Even more so, S100-A9 can directly activate platelets and promote procoagulant functions [163]. Data regarding S100-A9 in ME/CFS cohorts is scarce, and hence requires investigation, especially since it plays important roles in immune and vascular function. With regards to SARS-CoV-2, S100-A9 is increased during infection [164, 165].

### A comment on viral proteins

With regards to viral involvement, a study published in late 2022 discovered signs of active human herpesvirus 6 (HHV6) and Epstein-Barr Virus (EBV) in neurological tissue from deceased ME/CFS patients [6], thereby supporting previous hypotheses implicating herpesviruses in this condition [3, 5]. A more recent study also highlighted the presence and role of active herpes infection in a much larger cohort [7], as have other studies in the past [166–168]. There are also indications that viral reactivation of these herpesviruses is central to ME/CFS and Long COVID pathology [6, 169]. However, because much of the human population harbors latent herpesviruses (and indeed *Mycobacterium tuberculosis* and *Helicobacter pylori* and other dormant bacteria) without overt disease, the specific mechanisms by which their activity may contribute to ME/CFS pathology requires further study.

Our only significant DIA LC-MS/MS findings related to herpesviruses is the downregulation of protein UL29 from HHV6-H in the ME/CFS group. This is not conclusive, and most likely reflects the difficulty of detecting low-abundance proteins in plasma samples with a high dynamic range, especially when using global, untargeted proteomics approaches. Future studies aiming to confidently detect viral proteins should aim to decrease the dynamic range within plasma samples if doing DIA analyses, or perform targeted proteomics experiments. We also noticed that several viral proteins were phosphorylated; planning an experiment with this is in mind might offer information about viral activity.

### Conclusion

Identification of potential ME/CFS biomarkers is imperative for improved diagnosis, mechanism elucidation, and clinical care. Using DIA LC-MS/MS we show a significant, differential expression of proteins in PPP samples from ME/CFS and healthy individuals, involving the coagulation system, endothelium, and immune system.

Significant increases in TSP-1 and PF4, and a significant decrease in PROS suggest that the coagulation system is dysregulated in the present ME/CFS cohort. Our present and previous data [8] point to a procoagulant profile in ME/CFS, revolving around platelet hyperactivity and potentially a dysregulated endothelium. Related and recent studies are in accord with these inferences [11–14, 16, 17, 22, 23]. Incidentally, Long COVID suffers benefit from anticoagulant and antiplatelet therapy [170, 171]; further research is required to determine if this form of therapy will benefit ME/CFS patients exhibiting clotting pathology and thrombotic endothelialitis.

Beyond the coagulation system, our data further support immunological dysfunction in ME/CFS, including alterations in the complement system, as well as increases in LF and S100-A9. Deficits in the complement system, including a downregulation of the complement proteins that constitute the membrane-attack complex, could be related to viral infection and immune dysregulation in ME/CFS [3, 26], and deserves further study.

Our results highlight physiological systems – namely the cardiovascular, coagulation, and immune system – and proteins that require further research with regards to their contribution to the pathogenesis of ME/CFS, symptom manifestation, and biomarker potential. Furthermore, individuals from the ME/CFS study population were not diagnosed with diabetes mellitus, and hence this study gives insight into the thrombotic and cardiovascular risk associated with ME/CFS individuals also affected by diabetes mellitus.

### Study limitations

A major limitation of using DIA LC-MS/MS on samples that contain a large dynamic range is the detection and quantitation of low abundance proteins, such as herpesvirus proteins (not to mention the computational power and efficiency needed to identify and annotate each peptide detected). Future studies can aim to decrease the dynamic range when assessing viral proteins via this method, or employ targeted proteomics techniques (for the sake of identifying low-abundance proteins, as well as corroborating what was identified in the present, untargeted experiment).

Furthermore, a similar approach will benefit from a larger sample size and a further subdivision of the study groups by gender. The inclusion of a study population that experiences a similar lifestyle to that of ME/CFS individuals, i.e. a bed-bound population without ME/CFS, such as post-bone fracture patients, will also be beneficial. Another important point to raise is the influence of pre- and post-menopausal physiology in the female population [172, 173], which was not accounted for in our recruitment process.

## Electronic supplementary material

Below is the link to the electronic supplementary material.

Supplementary Material 1

Supplementary Material 2

## Data Availability

Supporting proteomics data is available on request.
