# Supplementary material for: Data-independent LC-MS/MS analysis of ME/CFS plasma reveals a dysregulated coagulation system, endothelial dysfunction, downregulation of complement machinery
Source: Cardiovasc Diabetol. 2024 Jul 16;23:254. doi: 10.1186/s12933-024-02315-x (PMC11253362; doi:10.1186/s12933-024-02315-x)
Supplement: Supplementary file 2 — Supplementary Material 2 [file 12933_2024_2315_MOESM2_ESM.docx]

**Platelet Factor 4 (355-555 m/z)**

**
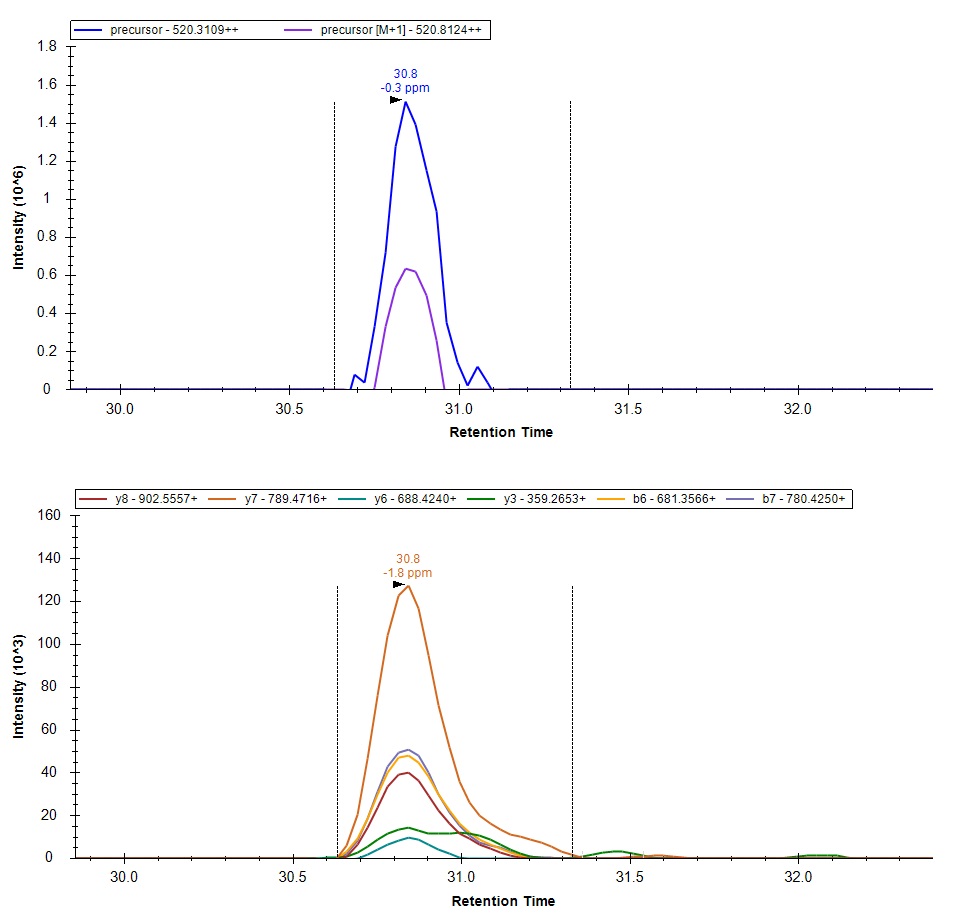
**

**Protein S100-A9 (555-755)**

**
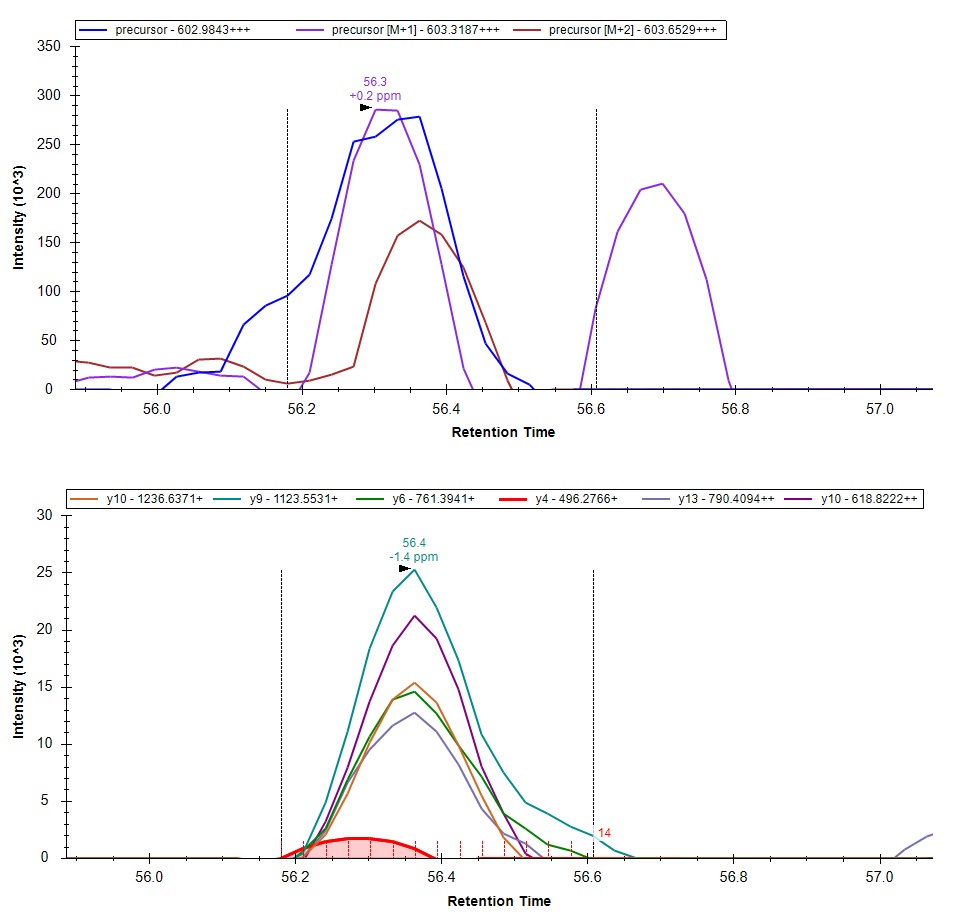
**

**Protein S100-A9 (755-955 m/z)**

**
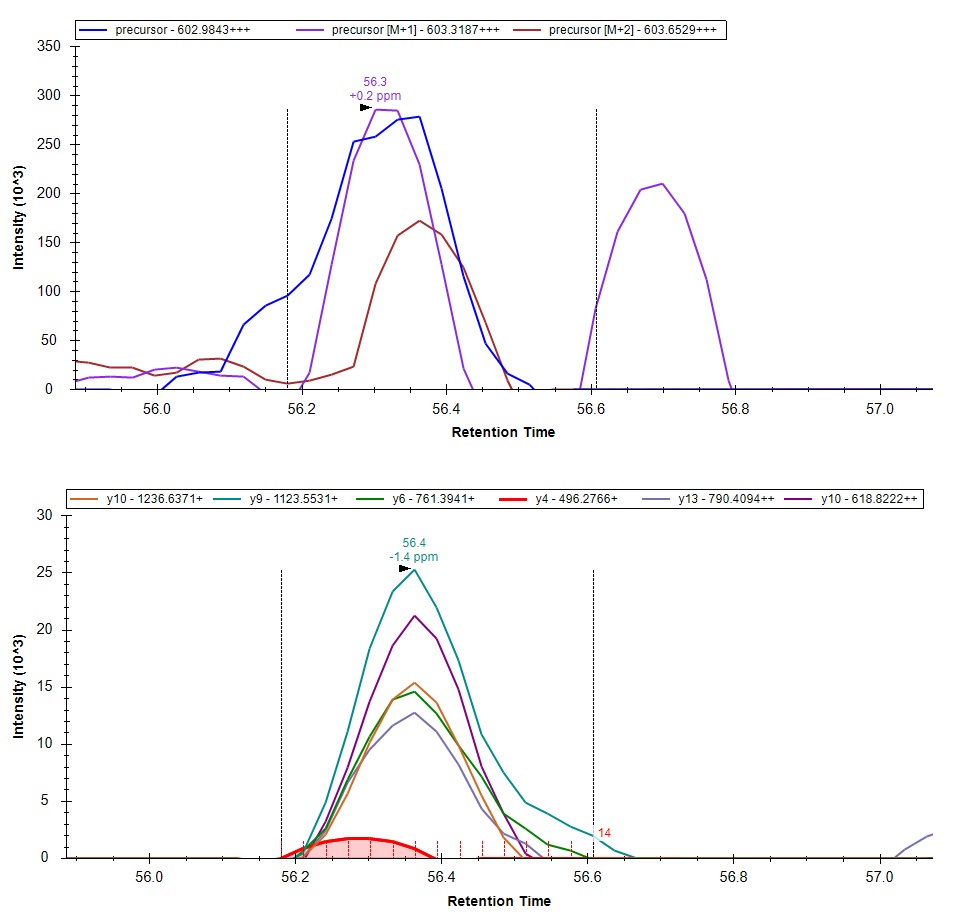
**

**5'-AMP-activated protein kinase subunit gamma-3**


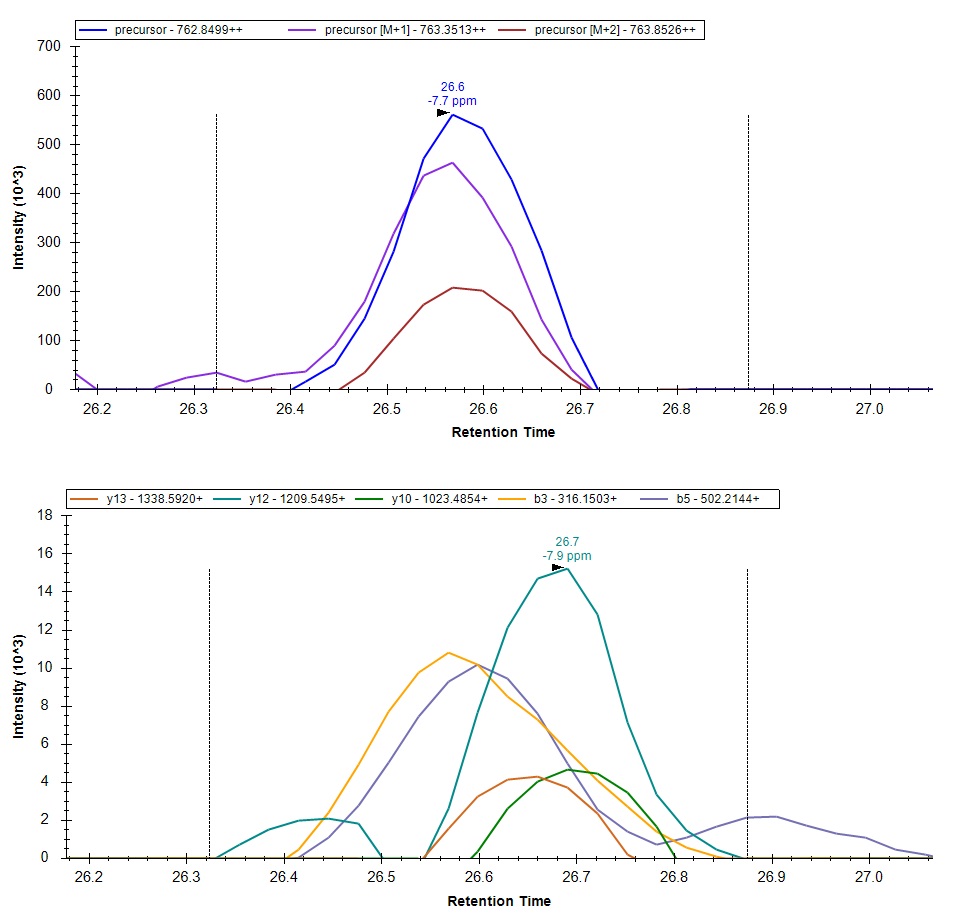


**Apolipoprotein C (755-995 m/z)**

**
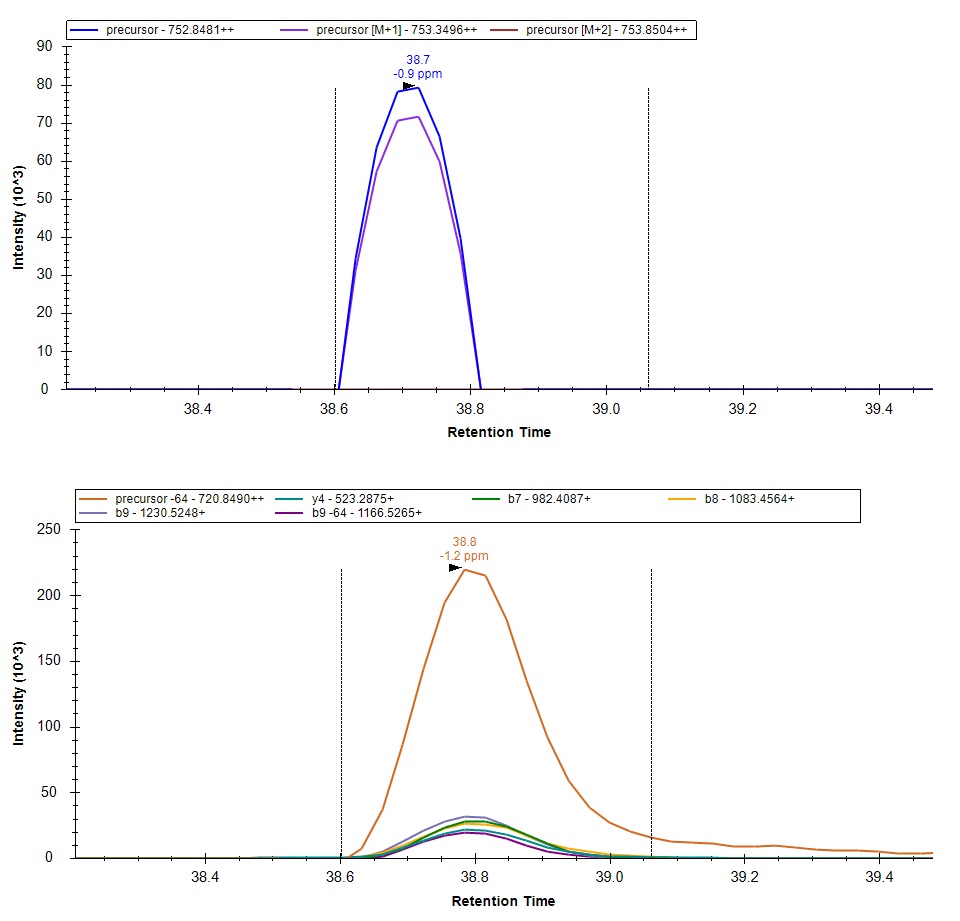
**

**Bargin (555-755 m/z)**

**
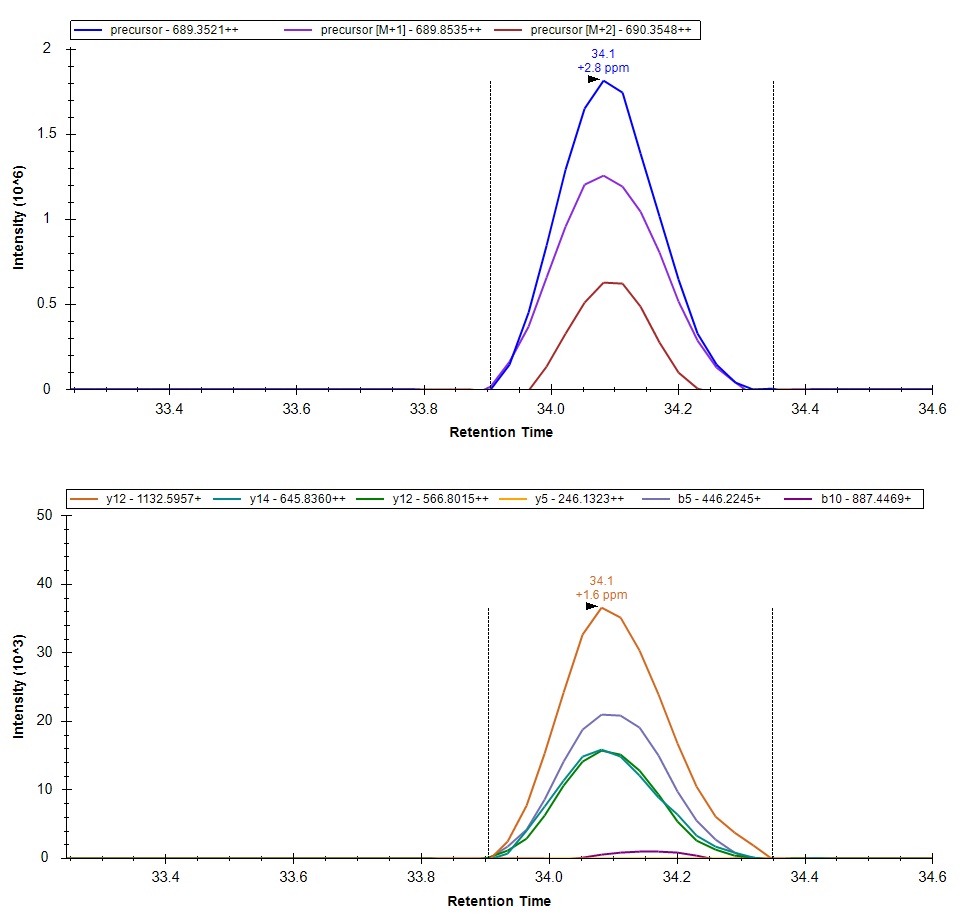
**

**E3 ubiquitin-protein ligase RNF213 (755-955 m/z)**


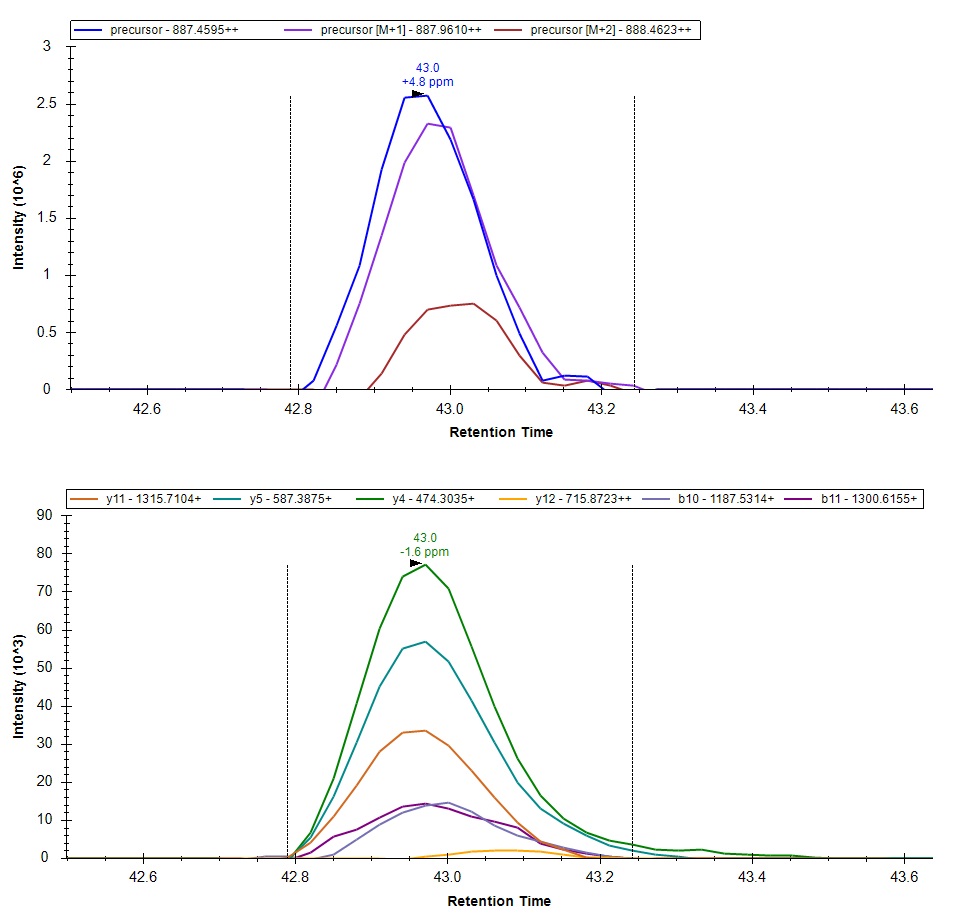


**Extended synaptotagmin-2 (755-955 m/z)**


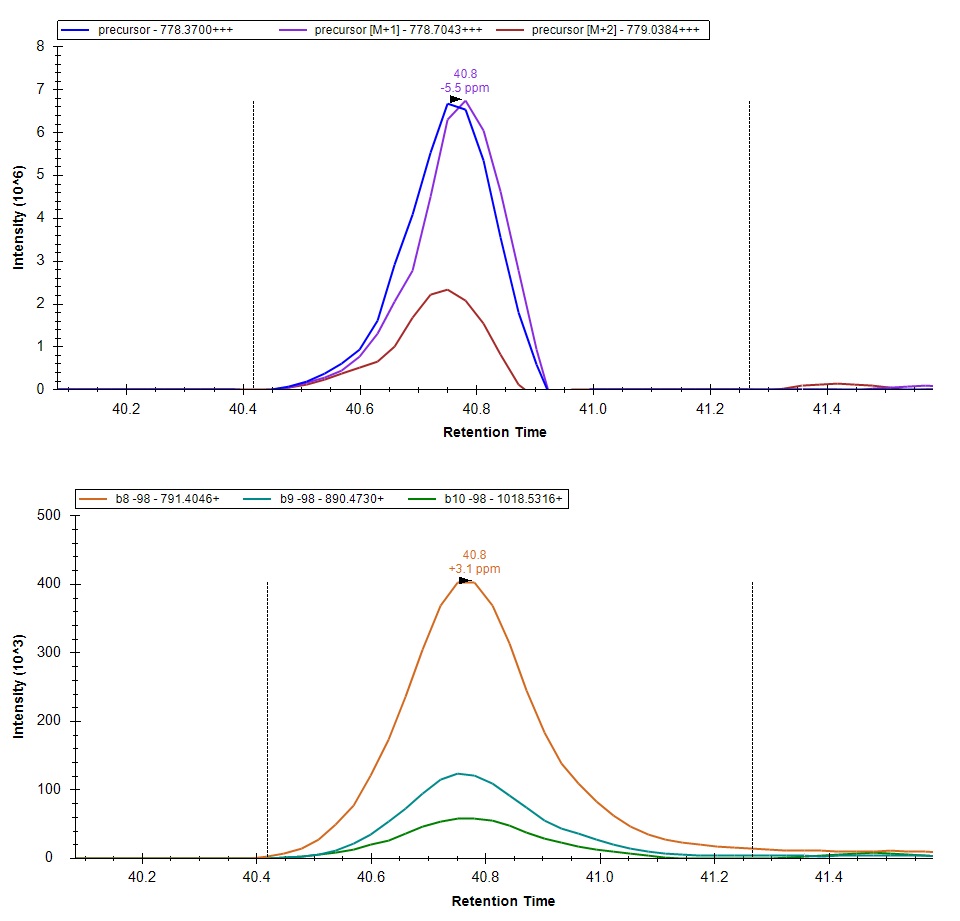


**Glyceraldehyde-3-phosphate dehydrogenase (555-755 m/z)**


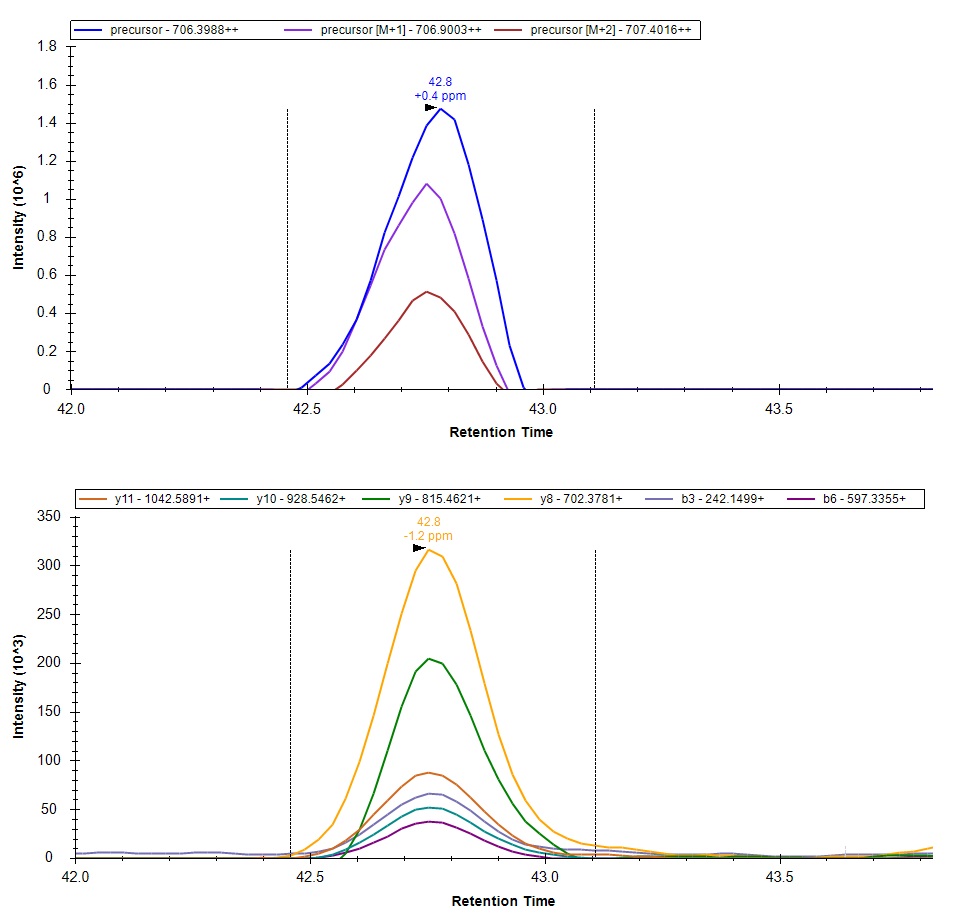


**Protein UL29 (HHV6-H) (355-555 m/z)**


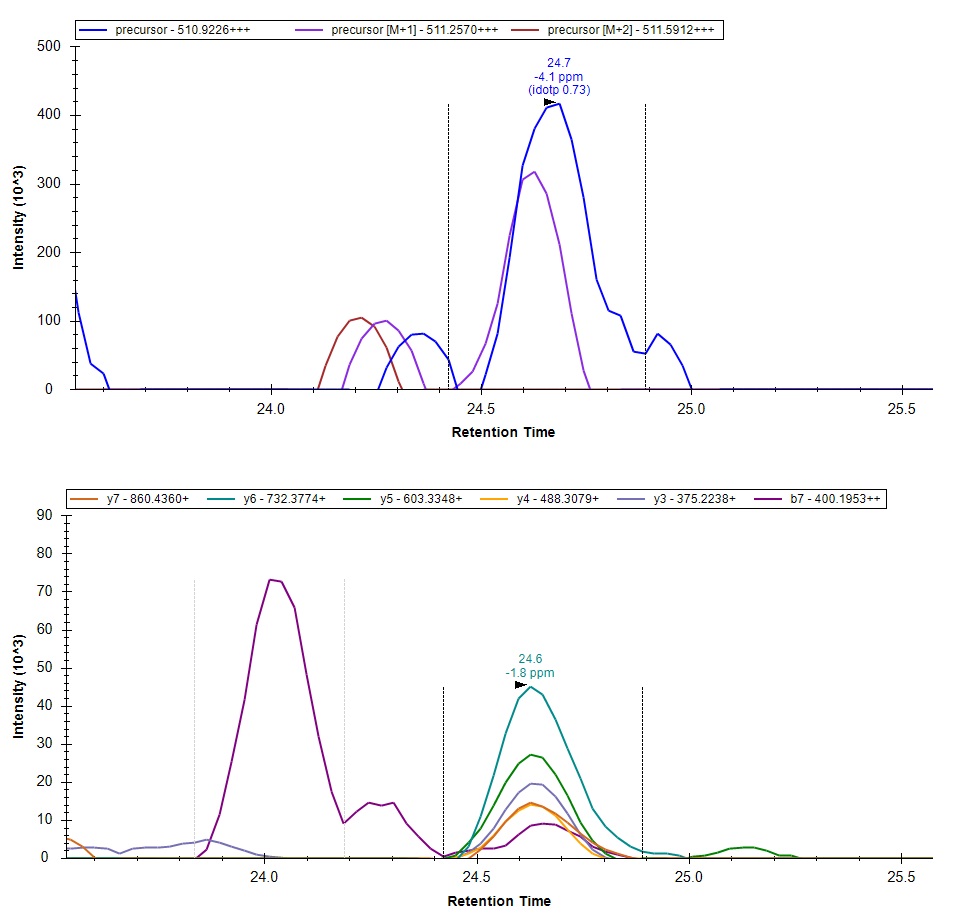


**Kinectin (355-555 m/z)**


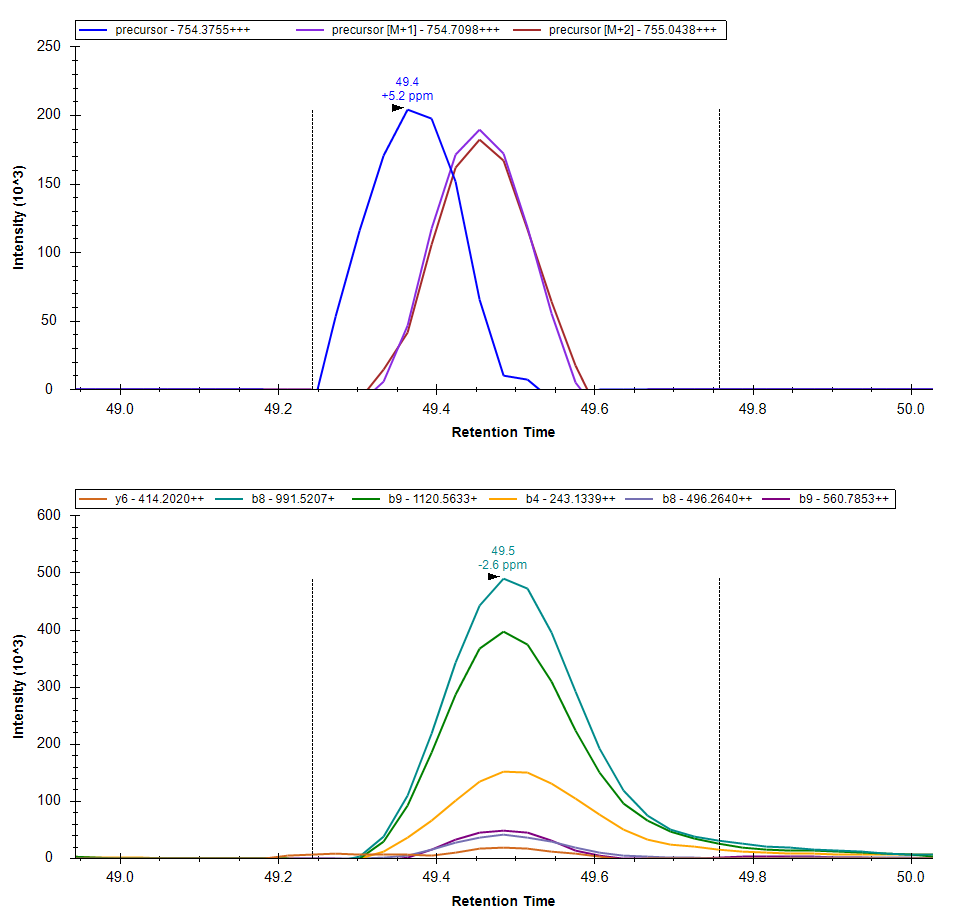


**Voltage-dependent P/Q-type calcium channel subunit alpha-1A (755-955 m/z)**

**
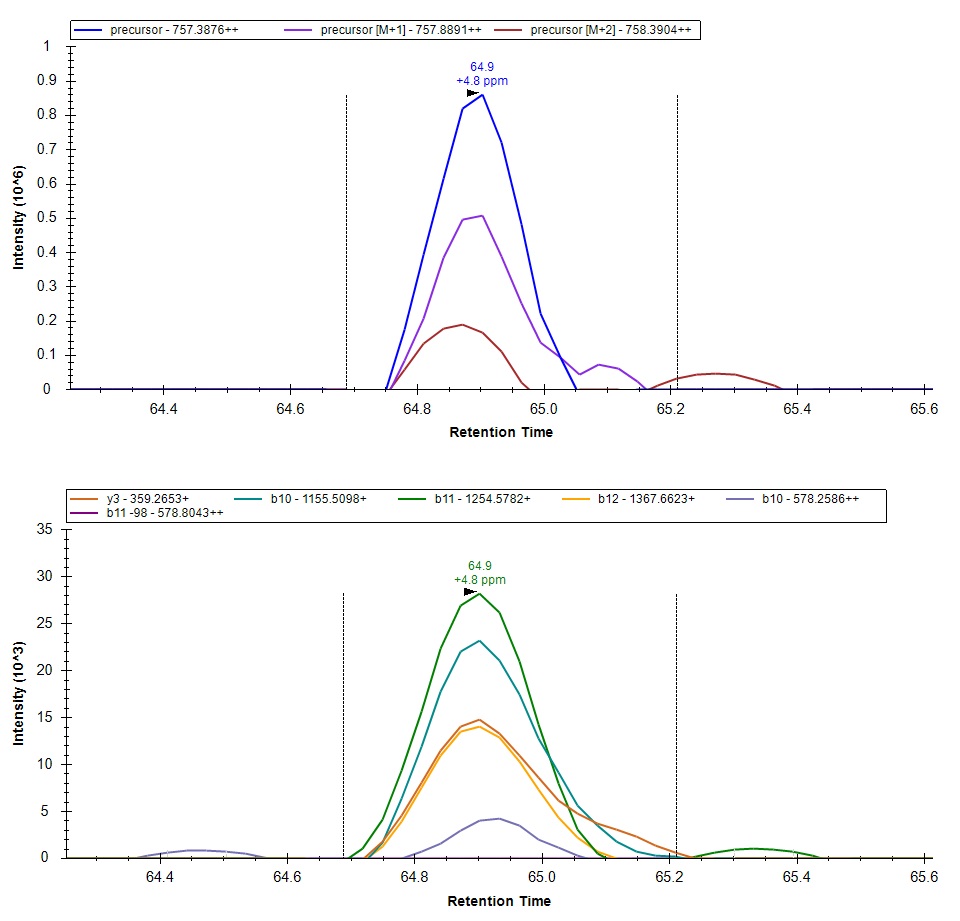
**
